# Emergent balloon pulmonary angioplasty in a patient with central‐type chronic thromboembolic pulmonary hypertension with cardiac shock

**DOI:** 10.1002/ccr3.6738

**Published:** 2022-12-13

**Authors:** Hiroto Tamura, Shinobu Hosokawa, Kenichiro Yuba

**Affiliations:** ^1^ Department of Cardiology Tokushima Red Cross Hospital Tokushima Japan

**Keywords:** chronic thromboembolic pulmonary hypertension, emergent balloon pulmonary angioplasty, pulmonary endarterectomy, pulmonary hypertension

## Abstract

The standard treatment of chronic thromboembolic pulmonary hypertension (CTEPH) is pulmonary endarterectomy (PEA); however, it may be unsuitable in the presence of comorbidities such as cardiac shock and severe hypoxia. We describe a successful case of emergent balloon pulmonary angioplasty performed before PEA in a patient with deteriorating central‐type CTEPH.

## Graphical abstract

An emergent balloon pulmonary angioplasty could be an alternative option before conventional pulmonary endarterectomy in a patient with deteriorating central‐type chronic thromboembolic pulmonary hypertension.

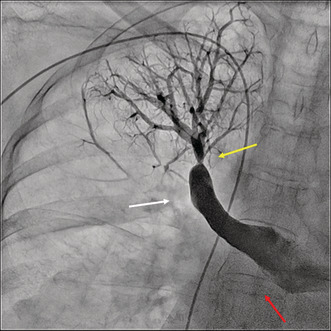

## 1 INTRODUCTION

Chronic thromboembolic pulmonary hypertension (CTEPH) is diagnosed based on a mean pulmonary artery pressure (PAP) higher than 20 mmHg and associated symptoms with signs of organized pulmonary thrombus despite more than 3 months of effective anticoagulation.^1^ It results in severe hypoxia and right heart failure. The gold standard treatment for CTEPH is pulmonary endarterectomy (PEA)^1^; however, a severe condition sometimes makes it impossible to perform.^2^ Balloon pulmonary angioplasty (BPA), an alternative treatment for organized thrombotic lesions of the peripheral pulmonary artery, has shown good progress in the case of inoperable CTEPH.^3^ Few reports have shown good results of emergent BPA in exacerbating central‐type CTEPH.^2^ Herein, we report a successful case of emergent BPA in a patient with central‐type CTEPH with cardiac shock.

## 2 CASE PRESENTATION

**FIGURE 1:**
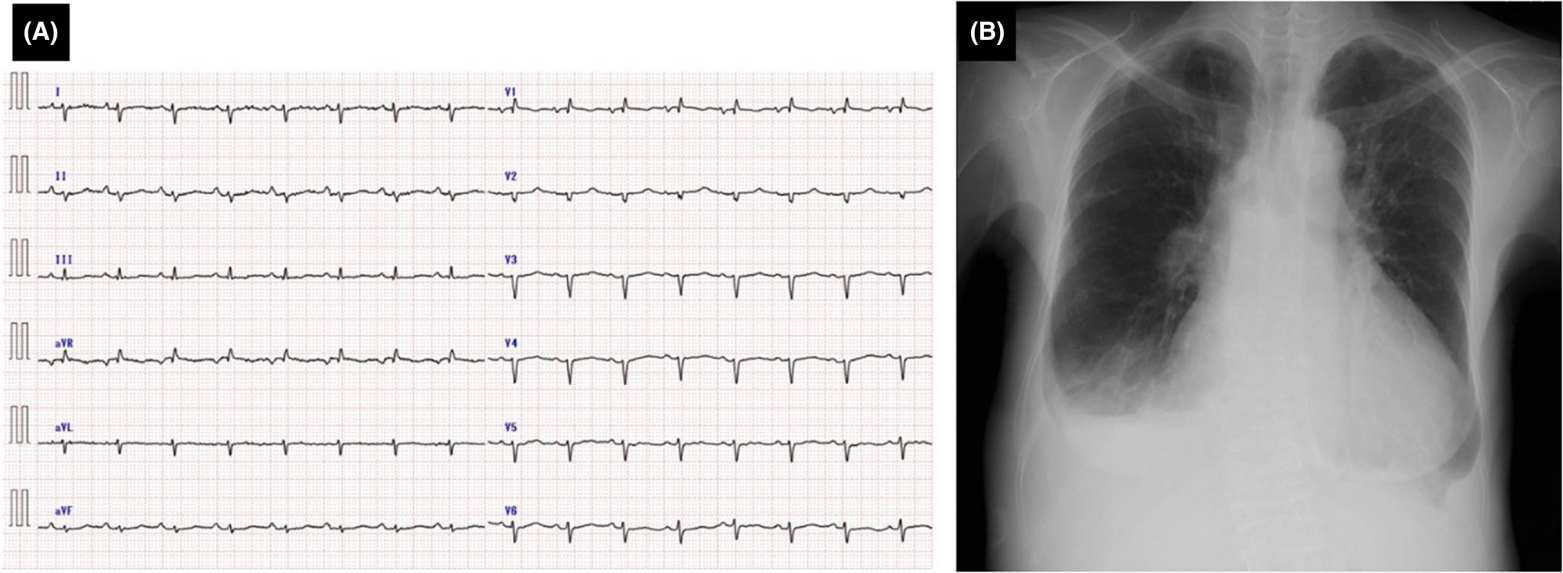
(A) 12‐lead resting electrocardiography, (B) Chest radiography on admission.

**FIGURE 2:**
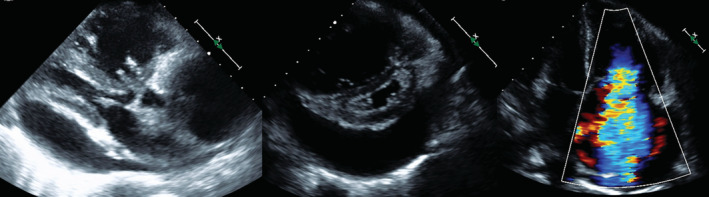
Echocardiography revealing dilatation of the right ventricle, severe tricuspid regurgitation with a flattened interventricular septum, and moderate pericardial effusion.

**FIGURE 3:**
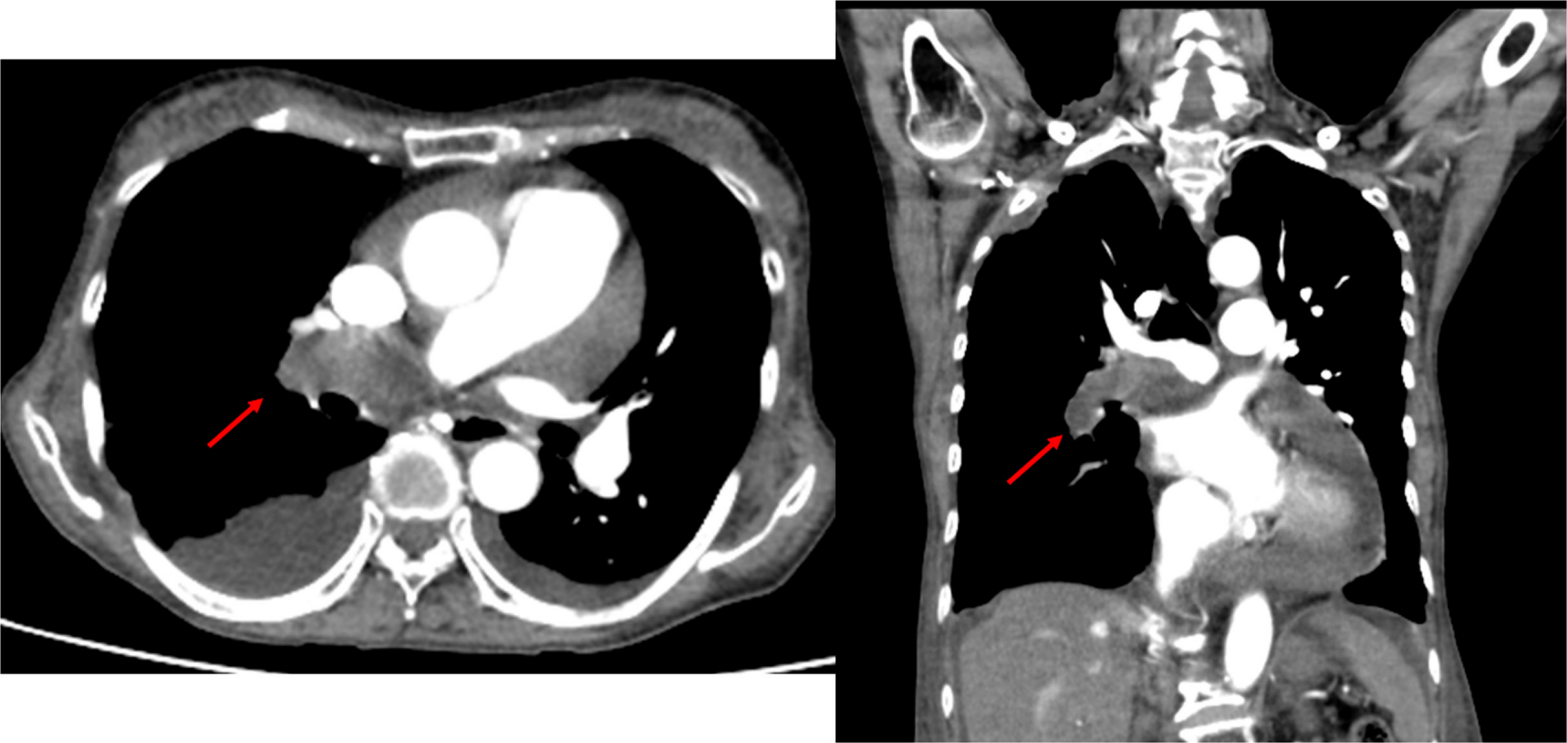
Thoracic contrast‐enhanced computed tomography showing a space‐occupying lesion, considered to be a pulmonary thrombus (red allow).

A 65‐year‐old woman experienced dyspnea 5 months before admission. Two months prior, her dyspnea started worsening, and lower leg edema developed. One month prior, her body weight increased by approximately 15 kg. She experienced intolerable respiratory discomfort and was referred to our emergency room. The patient's height and weight were 166 cm and 60 kg, respectively. On admission, her blood pressure was 109/71 mmHg; pulse rate, 91/min; oxygen saturation, 95% in room air; and body temperature, 36.5°C. The World Health Organization (WHO) functional class was III. A pansystolic murmur was heard at the cardiac apex. Additionally, respiratory rales were heard, and the lower limbs had severe edema. Electrocardiography showed a sinus rhythm and an incomplete right bundle branch block. Chest radiography presented obvious cardiomegaly and bilateral pleural effusions (Figure 1). Echocardiography revealed the dilatations of the right atrium and ventricle, severe tricuspid regurgitation with a flattened interventricular septum, a dilated inferior vena cava without respiratory change, and moderate pericardial effusion. The peak tricuspid regurgitation pressure gradient (TRPG) reached 75 mmHg. These findings suggested severe pulmonary hypertension (PH) (Figure 2). Laboratory findings revealed that the hemoglobin level was 13.8 g/dl; white blood cell count, 8520/μl; serum creatinine level, 0.63 mg/dl; D‐dimer level, 2.4 μg/ml; and brain natriuretic peptide (BNP) level, 883 pg/ml. Thoracic contrast‐enhanced computed tomography (CT) was performed because of severe PH. A space‐occupying lesion was recognized in the right mediastinum (Figure 3). Initially, the emergency physician suspected it to be a tumor and referred her to a surgeon. Positron emission tomography‐CT presented low standardized uptake values of the lesion, and the presence of a neoplastic lesion was ruled out. Therefore, the lesion was considered to be a pulmonary thrombus. She was diagnosed with acute pulmonary embolism and referred to our department. Initially, as there was a risk of cardiac tamponade, we performed pericardiocentesis, and 450 ml of pericardial effusion was removed. Echocardiography showed reduced pericardial effusion; however, her condition did not improve. Heparinization was initiated for acute pulmonary embolism. On the third day of hospitalization, oxygenation deteriorated and the severity of right heart failure increased rapidly. Saturation decreased to 90% (reservoir mask 10 L), and non‐invasive positive pressure ventilation was administered. The systolic pressure fell to 80 mmHg, and the pulse rate increased to 120/min. Catecholamine administration was indispensable for the management of cardiac shock. We administered 1,200,000 Units of recombinant tissue plasminogen activator (Cleactor, Eisai Co. Ltd.). However, thrombolysis did not occur. Hence, we considered the space‐occupying lesion to be an organized thrombus. The WHO functional class aggravated to IV. Emergent right heart catheterization (RHC) and pulmonary angiography (PAG) were performed.

**FIGURE 4:**
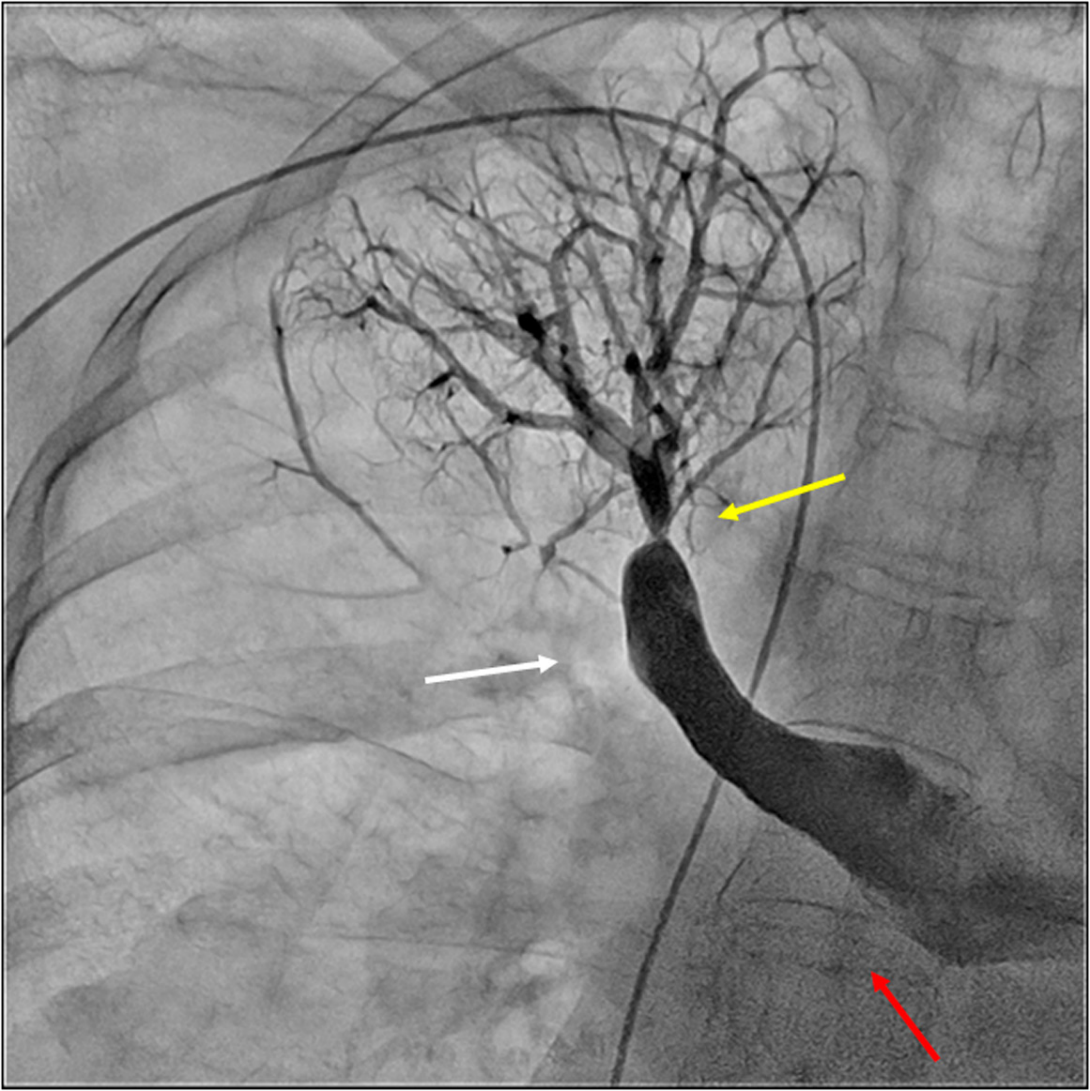
Pulmonary angiography showing a pouch defect in the right A3 segment (white arrow), a pouch defect in the right basal segment of the lower lobe (red allow), and ring‐like stenosis in the right A1 segment (yellow arrow).

**FIGURE 5:**
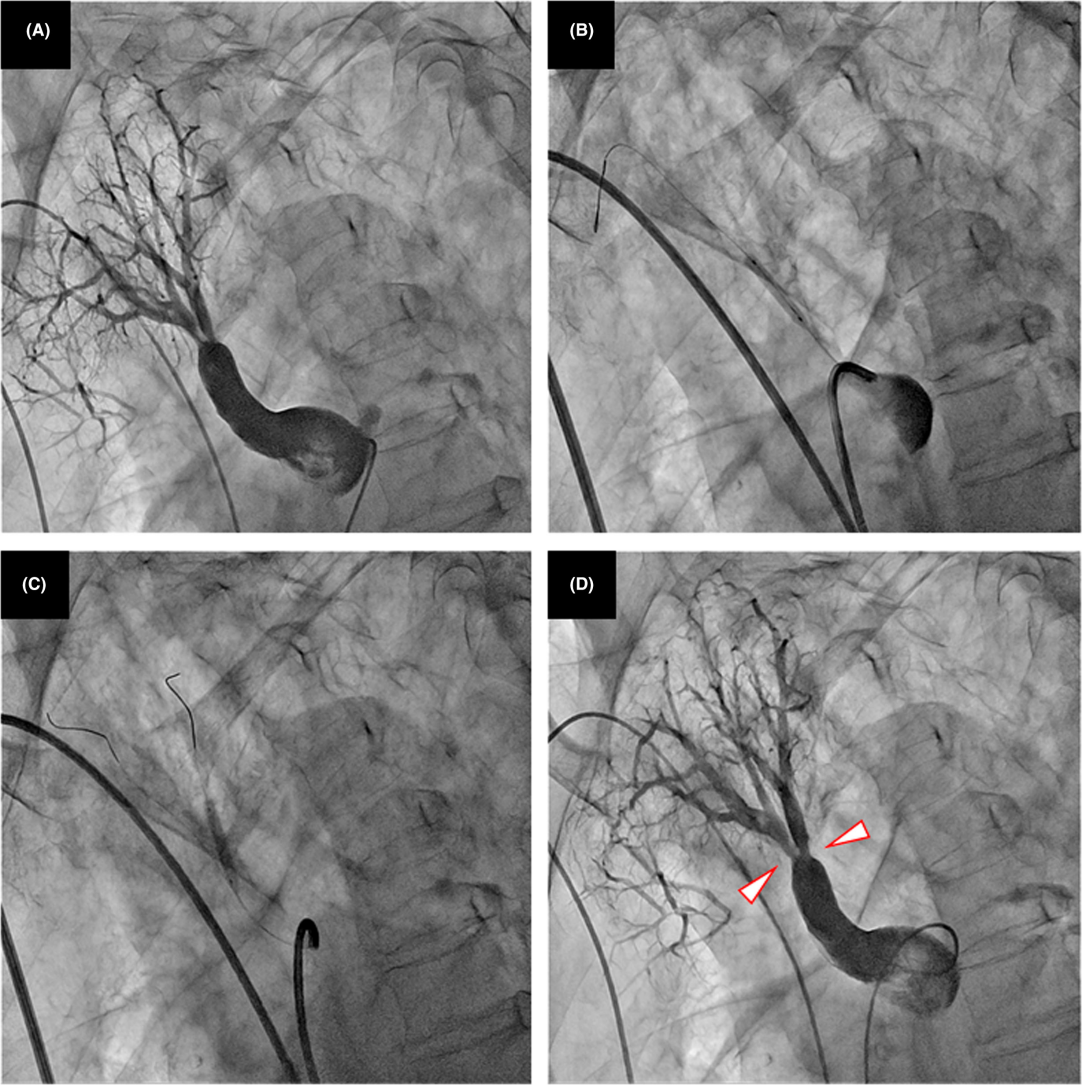
Pulmonary angiography in A1. (A) Before balloon pulmonary angioplasty; (B, C) Dilatation of the ring‐like stenosis lesion with a 4‐mm balloon; (D) Enlargement of the pulmonary artery after balloon pulmonary angioplasty (arrowheads).

We could not convey Swan‐Ganz catheter to the main pulmonary artery and abandoned RHC. PAG showed an organized thrombotic pouch defect in the right A3 segment and right basal segment of the lower lobe, and multiple web lesions or ring‐like stenosis in the other multiple lesions (Figure 4). Although the mean PAP could not be measured precisely, these findings were comparable with those of CTEPH; the main lesion was undoubtedly in proximal pulmonary artery. The standard treatment for this type of CTEPH was PEA; however, the condition of the patient was critical with cardiac shock and severe hypoxia. Performing surgical treatment to rescue this patient seemed impossible. Therefore, we decided on emergent BPA for the relatively simple lesions. We carefully crossed two lesions in the A1 segment with 0.014‐inch wires (Chevalier Universal, NIPRO; VASSALLO Floppy, Cordis) and dilated each lesion with a 4‐mm non‐compliant balloon (Coyote NC, Boston Scientific) (Figure 5). There was a concern of vessel injury due to high pulmonary pressure. Hence, the balloon was dilated very slowly. The pulmonary flow improved after dilation and better pulmonary venous return was obtained. BPA was terminated without any complications. After emergent BPA, the progressive dyspnea and severity of right heart failure improved significantly. On the fifth day of hospitalization, riociguat was initiated for CTEPH. On the ninth day of hospitalization, warfarin was initiated instead of heparin. Catecholamine was withdrawn, her weight dropped to 45 kg, and her lower leg edema disappeared. The BNP level improved to 236 pg/ml after BPA. The anti‐centromere antibody level elevated to 1280 times, and Raynaud's phenomenon appeared. Therefore, CREST syndrome was suspected; however, the diagnostic criteria were not met. The cause of pericardial effusion was not clear. On the 28th day of hospitalization, RHC and PAG were reinspected. The RHC result revealed a PAP of 67/27 mmHg with 42 mmHg of mean pressure; pulmonary arterial wedge pressure (PAWP), 12 mmHg; cardiac output (CO), 3.47 L/min; and pulmonary vascular resistance (PVR), 8.65 Wood units (Table 1). PAG revealed unchanged thrombotic lesions despite the continuation of warfarin. Although warfarin was not continued for at least 3 months, considering these findings, the patient was diagnosed with central‐type CTEPH. PH persisted and we recommended PEA as the treatment for complete recovery. On the 67th day of hospitalization and rehabilitation, she was transferred to a different hospital for PEA.

**TABLE 1:** Result of right heart catheterization.

PAWP	12 mmHg
PAP(s/d/m)	67/27/42 mmHg
CO	3.47 L/min
PVR	8.65 Wood units

## 3 DISCUSSION

Chronic thromboembolic pulmonary hypertension is defined as Group 4 PH.^1^ The treatment strategy has been extended recently to anticoagulation, pulmonary vasodilators (riociguat and selexipag), BPA, and PEA.^1^, ^4^, ^5^ These current advances provide a good prognosis, and the 5‐year survival rate is 92.0%, which is extremely good compared to those of other types of PH.^3^ Generally, this disease has a chronic course; however, in some cases, the condition of the patient deteriorates, as in our case. Appropriate emergency rescue is required for better outcomes. The standard treatment for central‐type CTEPH remains PEA.^1^ In this case, PAG showed a massive organized thrombus in the right basal segment of the lower lobe. A surgical approach is generally recommended; however, it was unsuitable due to comorbidities such as cardiac shock and severe hypoxia. BPA is an endovascular therapy and a relatively less invasive alternative.^1^ However, the PAP increases due to complications such as pulmonary vessel injury and reperfusion pulmonary edema.^6^ Moreover, its severity is exacerbated by a high mean PAP.^6^ Therefore, under such critical conditions, BPA must be performed without any complications. Kawakami et al. established the angiographic lesion classification, prediction for success, and complication rate of BPA.^7^ The success rate of BPA for a totally occluded lesion was as low as 52.2%. However, the success rate of BPA in a ring‐like stenosis lesion or web lesion was as high as 100% or 98.7%, with a low rate of 1.6% or 2.2%, respectively, with complications. Because of the high risk to the pouch defect lesion, we decided to treat the ring‐like stenosis lesion, across where we could pass the wire safely. Dilation to several lesions in one session was required,^6^ but our purpose was the safe stabilization of hemodynamics. We treated the minimum number of lesions required to improve the condition sufficiently. This treatment strategy could rescue our patient with less risk. Tsuji et al. reported a successful case of emergent BPA for a patient with deteriorating proximal CTEPH.^2^ Emergent BPA could be an alternative option before conventional PEA for patients with deteriorating central‐type CTEPH patient. Simple lesions should be dilated with undersized balloons to minimize complications of the BPA procedure.

## AUTHOR CONTRIBUTIONS

**Hiroto Tamura:** Conceptualization; data curation; investigation; resources; visualization; writing – original draft; writing – review and editing. **Shinobu Hosokawa:** Investigation; methodology; project administration. **Kenichiro Yuba:** Resources; supervision; validation; visualization.

## FUNDING INFORMATION

None declared.

## CONFLICT OF INTEREST

The authors declare that there is no conflict of interest.

## ETHICAL APPROVAL

The study was published with written consent of the patient.

## CONSENT

Written informed consent was obtained from the patient for the publication of this case and the accompanying images.

## Data Availability

The data that support the findings of this study are available from the corresponding author upon reasonable request.
